# A camouflaged carcinoma and it's surgical encounter - A case report

**DOI:** 10.1016/j.ijscr.2025.112013

**Published:** 2025-10-07

**Authors:** Chintamani, Shagun Agarwal, Sakura Shrestha, Sonia Badwal, Tanushree Nahata, Kashish Arora

**Affiliations:** a Department of Surgical Oncology, Sir Ganga Ram Hospital, New Delhi, India; b Department of Histopathology, Sir Ganga Ram Hospital, New Delhi, India

**Keywords:** Basal cell carcinoma, Axilla, Non-melanoma skin cancer, Hedgehog pathway, Case report, Skin cancer in sun-protected areas

## Abstract

**Background:**

Basal Cell Carcinoma (BCC) is the most prevalent form of skin cancer, typically arising in sun-exposed areas, especially the head and neck. However, its occurrence in sun-protected regions, such as the axilla, is exceedingly rare and often leads to diagnostic challenges. This report highlights an uncommon presentation of axillary BCC, emphasizing the need for heightened clinical suspicion and histopathological confirmation in atypical locations.

**Case presentation:**

We report the case of a 58-year-old male presenting with a slowly enlarging left axillary mass over three years, associated with bluish discoloration and a small ulcer. Initial wedge biopsy suggested squamous cell carcinoma, prompting wide local excision with axillary lymph node dissection. Histopathology subsequently revealed nodular basal cell carcinoma with clear surgical margins and no nodal metastasis. The patient had an uneventful recovery and remains disease-free at one-year follow-up.

**Discussion:**

BCC rarely affects non-sun-exposed areas, and when it does, differential diagnosis becomes complex. Chronic irritation, immunosuppression, and genetic syndromes like Gorlin syndrome may contribute to pathogenesis. This case underscores the importance of considering BCC even in uncommon anatomical locations. A review of current literature and treatment modalities, including surgical excision, Mohs surgery, and targeted Hedgehog pathway inhibitors, is presented.

**Conclusion:**

BCC of the axilla is rare but should be considered in the differential diagnosis of chronic axillary lesions. Histopathology remains essential for accurate diagnosis. Early identification and appropriate management can lead to excellent outcomes even in atypical presentations.

## Highlights

• Axillary BCC is an extremely rare clinical entity.
• Misdiagnosed initially as squamous cell carcinoma.
• Wide local excision with lymph node dissection was performed.
• Histopathology confirmed nodular basal cell carcinoma.
• Clear margins and disease-free status at one-year follow-up.

## 1 Introduction

Melanoma is the most common skin cancer worldwide. Of the two Non- Melanoma Skin Cancers (NMSC), Squamous Cell Cancer (SCC) is the most common in Asians, Blacks, and Indians. However, Basal Cell Cancer (BCC) is the most common NMSC in the whites. This can be explained by the presence of Melanin pigment that provides protection against DNA damage incurred by Ultraviolet Radiation (UVR), responsible for carcinogenesis [1].

In the following case report, we present a patient with a peculiar malignancy of the axillary region and its appropriate management, followed by literature review.

## 2 Case

**Fig. 1:**
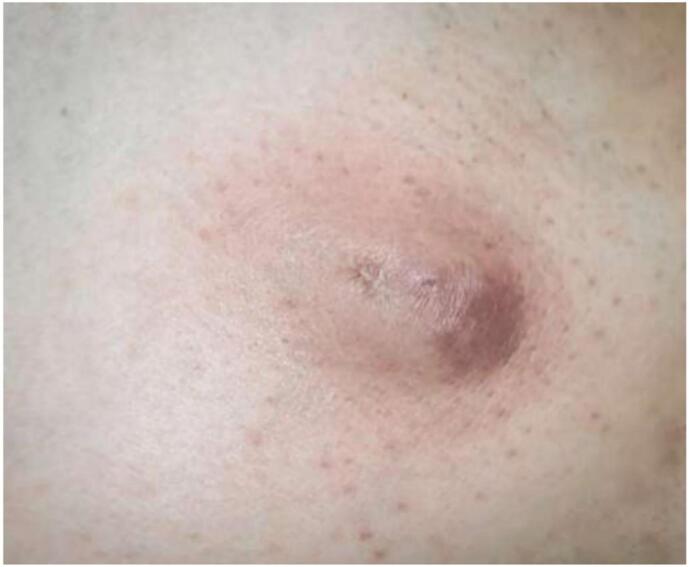
Eccentric ulcer over an axillary swelling.

**Fig. 2:**
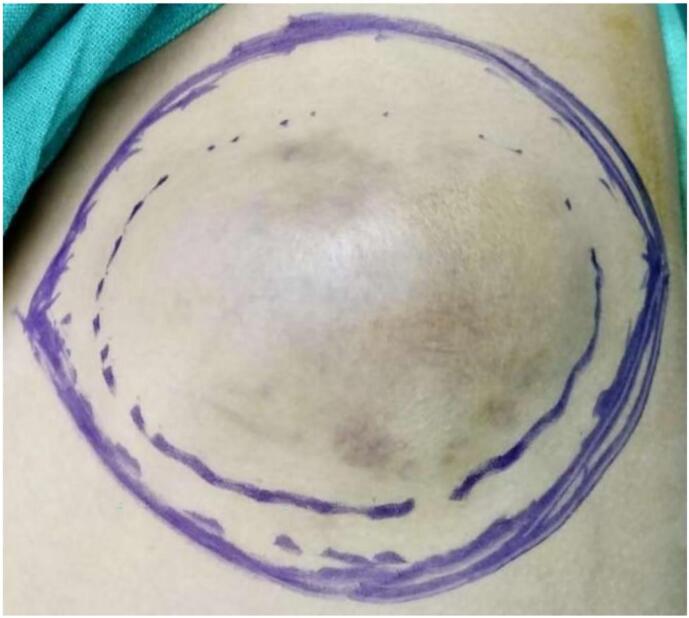
Bluish discoloration of the intact overlying skin.

58 years Gentleman presents with a swelling in Left Axillary region for 3 years, gradually progressive in size. There was some skin change noticed in the form of reddish and bluish discoloration over the course of these 3 years. He is a resident of Uttar Pradesh region of India, and clerk by profession. No history of any surgery/Ionizing radiation exposure/ arsenic or coal tar exposure in the past. On examination, a 4 × 3 cm swelling could be noticed in the left axilla with bluish discoloration of the intact overlying skin, apart from an eccentric ulcer of 0.5 × 0.5 cm. The swelling was attached to the skin but free from the underlying Latissimus Dorsi (LD) and Pectoralis Major (PM) Muscle. Multiple axillary lymph nodes were palpable, discrete, non-tender, mobile- Palpable lymph nodes ranged from 1 cm to 2.5 cm in diameter (Figs. 1 and 2).

**Fig. 3:**
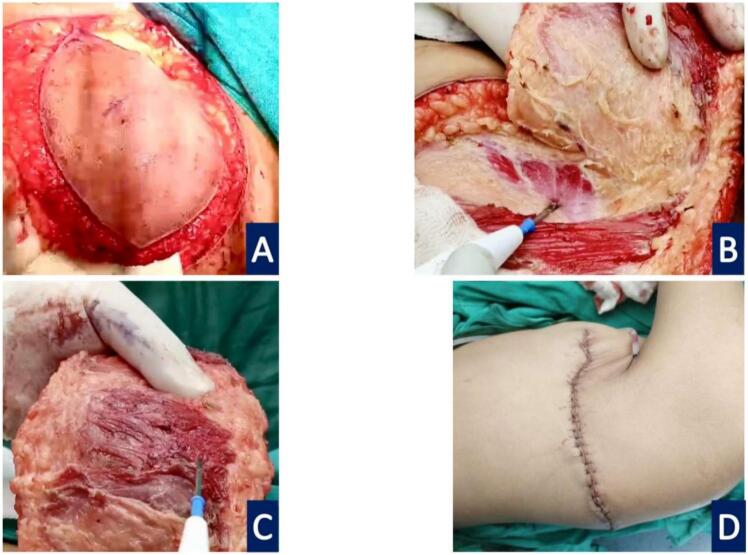
Intraop findings. A- Excision margins. B- Tumor bed. C- Pectoralis muscle chunk as deep resected margin. D- Primary closure.

A wedge biopsy was performed, that suggested: Squamous Papilloma with Malignant Changes, probably Squamous Cell Carcinoma. Hence a plan for Wide Local Excision (1 cm margin) with 3-dimensional clearance and axillary dissection was made. The patient underwent said procedure, and intraoperatively the underlying PM chunk was excised en masse, leaving the LD intact. There were 3 enlarged axillary nodes also present, which were excised and Levels I, and II axillary lymph nodes dissected. The wound was primary closed (Fig. 3).

**Fig. 4:**
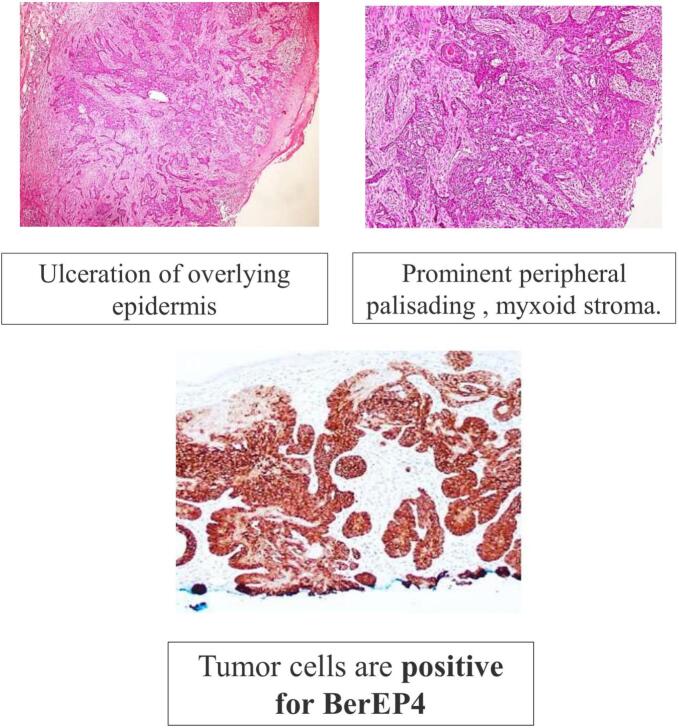
Histopathology Images (H&E, 40x) and Special Stain slide.

Patient had an uneventful recovery. The post-operative histopathology findings were suggestive of Basal cell carcinoma. All nodes were negative for metastasis. Histopathological examination confirmed tumor-free margins of ≥5 mm in all directions (Fig. 4).

The patient is on regular follow up with healed scar and no evidence of recurrence or distant metastasis.

## 3 Discussion

**T**he work has been reported in line with the SCARE criteria [8].

### 3.1 Epidemiology and risk factors

BCC is most common in fair-skinned populations, particularly in sun-exposed regions such as the head and neck. However, its occurrence in sun-protected sites like the axilla is rare. The axilla is considered a sun-protected area due to its anatomical location. Moreover, in the Indian context—especially for male office workers like our patient—this region remains consistently covered by clothing due to social and environmental factors, making direct sunlight exposure unlikely.

Factors contributing to BCC in these regions include advancing age, chronic irritation, immunosuppression, and genetic predispositions such as Gorlin syndrome and xeroderma pigmentosum [1].

Phenotypic factors, including fair skin, light-colored eyes, and a propensity to burn rather than tan, also increase susceptibility. Additionally, aberrant activation of the Hedgehog signaling pathway, driven by mutations in the patched 1 (PTCH1) and smoothened (SMO) genes, plays a critical role in BCC pathogenesis. These mutations disrupt normal cell cycle regulation, leading to uncontrolled cellular proliferation [2].

### 3.2 Clinical features

BCC commonly presents as a slowly growing lesion, with subtypes including [3]:

1. Nodular BCC: Pearly, translucent papules with telangiectasia.
2. Superficial BCC: Erythematous, scaly plaques often misdiagnosed as eczema.
3. Morpheaform BCC: Indurated, scar-like lesions with poorly defined margins, making them more aggressive.
4. Pigmented BCC: Lesions containing melanin, mimicking melanoma.

In this case, the lesion's axillary location and bluish discoloration presented a diagnostic challenge, emphasizing the need for histopathological confirmation.

### 3.3 Diagnosis

While the diagnosis of BCC is often clinical, histopathological examination is mandatory for atypical presentations. Dermoscopy aids in identifying characteristic features such as arborizing vessels and blue-gray ovoid nests [4]. In this case, wedge biopsy was instrumental in confirming the diagnosis. However a wide excision with 3- D clearance gave the actual and complete diagnosis postoperatively along with cure.

### 3.4 Management

Treatment goals include complete tumor removal while preserving function and aesthetics. Surgical excision remains the gold standard, ensuring clear margins. Mohs micrographic surgery is preferred for high-risk or recurrent lesions. Non-surgical options, such as topical therapies (imiquimod, 5-fluorouracil), cryotherapy, photodynamic therapy, and radiation therapy, are reserved for specific subtypes or inoperable cases [5].

For advanced or metastatic BCC, targeted therapies like vismodegib and sonidegib, which inhibit the Hedgehog pathway, have demonstrated efficacy [6].

### 3.5 Recent advances

Emerging therapies targeting molecular pathways, such as immune checkpoint inhibitors, are being explored in clinical trials. Imaging modalities like optical coherence tomography and confocal microscopy are improving diagnostic accuracy and treatment planning, particularly for challenging cases like this one [2].

### 3.6 Prognosis and prevention

The prognosis for BCC is excellent with early detection and appropriate treatment. Preventive strategies include minimizing UV exposure, using broad-spectrum sunscreen, and regular skin examinations, particularly for high-risk individuals.

## 4 Conclusion

This case underscores the importance of considering BCC in the differential diagnosis of unusual axillary lesions, despite its rarity in sun-protected sites. While evolving clinical data suggest broader anatomical presentations of BCC, its occurrence in sun-protected areas like the axilla remains rare, with fewer than 30 cases reported in literature to date [7]. Thus, it remains an uncommon clinical presentation worth documenting. Histopathological confirmation and a multidisciplinary approach are critical for optimal outcomes. Ongoing research into targeted therapies and non-invasive diagnostic techniques holds promise for further advancements in the management of BCC.

## Author contribution

Chintamani- Initiation of the idea for case report, primary surgeon and responsible consultant for the patient.

Shagun Agarwal- Drafting of the manuscript.

Sakura Sreshtha- Coordination with the pathologist, Editing of the final Manuscript.

Sonia Badwal- Primary pathologist who diagnosed the disease, pathological images facilitation.

Tanushree Nahata- Patients dressings in post op period, arrangement of clinical details of the patient.

Kashish Arora- Organising the manuscript.

## Consent

Written informed consent was obtained from the patient for publication and accompanying images. A copy of the written consent is available for review by the Editor-in-chief of the journal on request.

## Ethical approval

This is a case report- exempt from ethical approval in our institution.

Institution name: Sir Ganga Ram Hospital, New Delhi, India.

## Guarantor

Chintamani and Shagun Agarwal.

## Research registration number

NA.

## Funding

None.

## Conflict of interest statement

None.
